# 4,4′-Di-4-pyridyl-2,2′-dithio­dipyrimidine

**DOI:** 10.1107/S1600536809022004

**Published:** 2009-06-17

**Authors:** Hai-Bin Zhu, Hai Wang, Lei Li

**Affiliations:** a School of Chemistry and Chemical Engineering, Southeast University, Nanjing, People’s Republic of China

## Abstract

In the title mol­ecule, C_18_H_12_N_6_S_2_, the C—S—S—C torsion angle is 96.12 (9)°. The dihedral angles between the pyridyl and pyrimidinyl rings are 16.7 (1) and 1.27 (9)°. In the crystal, inter­molecular π–π inter­actions between the aromatic rings [centroid–centroid distances = 3.888 (2) and 3.572 (1) Å] link mol­ecules into chains propagating in [011].

## Related literature

For related crystal structures, see: Ji *et al.* (2009 ▶); Higashi *et al.* (1978 ▶); Tabellion *et al.* (2001 ▶). For general background to heterocyclic disulfides, see: Horikoshi & Mochida (2006 ▶).
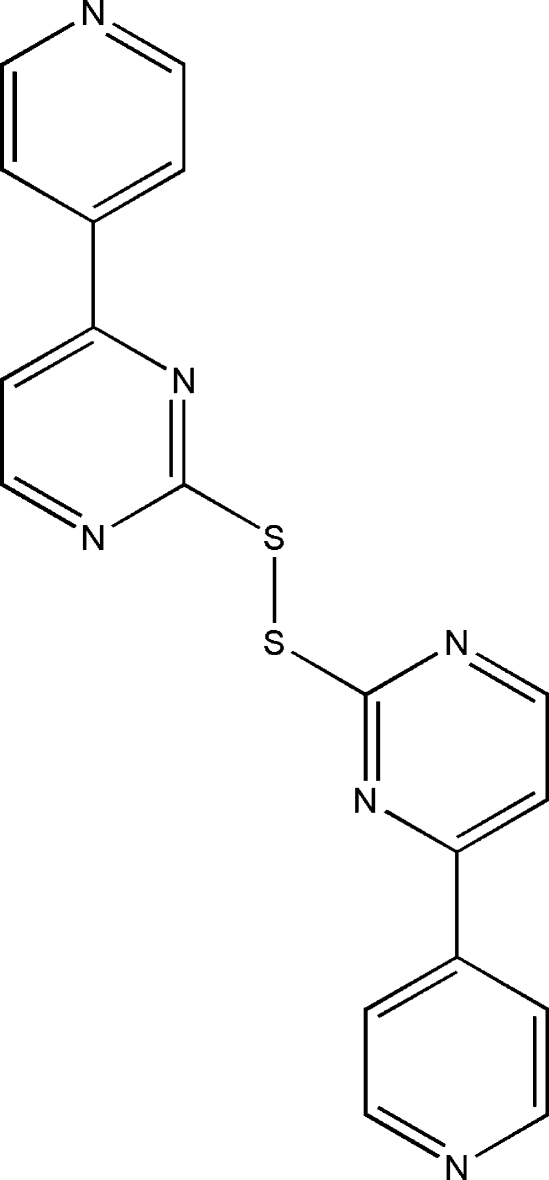


## Experimental

### 

#### Crystal data

C_18_H_12_N_6_S_2_
*M* *_r_* = 376.48
Triclinic, 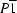

*a* = 9.1060 (8) Å
*b* = 9.3861 (9) Å
*c* = 10.9176 (10) Å
α = 84.228 (1)°
β = 74.926 (1)°
γ = 72.983 (1)°
*V* = 861.27 (14) Å^3^
*Z* = 2
Mo *K*α radiation
μ = 0.32 mm^−1^
*T* = 298 K
0.14 × 0.12 × 0.10 mm

#### Data collection

Bruker APEXII CCD area-detector diffractometer
Absorption correction: multi-scan (*SADABS*; Bruker, 2001 ▶) *T*_min_ = 0.884, *T*_max_ = 0.920 (expected range = 0.930–0.968)
5665 measured reflections
3982 independent reflections
3283 reflections with *I* > 2σ(*I*)
*R*_int_ = 0.093

#### Refinement

*R*[*F* ^2^ > 2σ(*F* ^2^)] = 0.056
*wR*(*F* ^2^) = 0.158
*S* = 1.12
3982 reflections
235 parameters
H-atom parameters constrained
Δρ_max_ = 0.63 e Å^−3^
Δρ_min_ = −0.50 e Å^−3^

### 

Data collection: *APEX2* (Bruker, 2007 ▶); cell refinement: *SAINT-Plus* (Bruker, 2007 ▶); data reduction: *SAINT-Plus*; program(s) used to solve structure: *SHELXS97* (Sheldrick, 2008 ▶); program(s) used to refine structure: *SHELXL97* (Sheldrick, 2008 ▶); molecular graphics: *SHELXTL* (Sheldrick, 2008 ▶); software used to prepare material for publication: *SHELXTL*.

## Supplementary Material

Crystal structure: contains datablocks I, global. DOI: 10.1107/S1600536809022004/cv2571sup1.cif

Structure factors: contains datablocks I. DOI: 10.1107/S1600536809022004/cv2571Isup2.hkl

Additional supplementary materials:  crystallographic information; 3D view; checkCIF report

## Figures and Tables

**Fig. 1.**
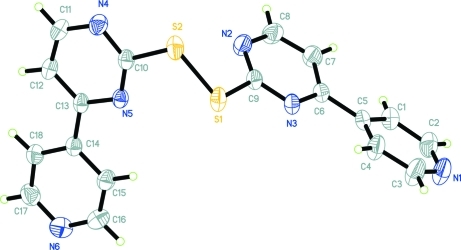
The molecular structure of the title compound showing the atomic numbering and 30% probability displacement ellipsoids.

## supplementary crystallographic information

### Comment

Heterocylic disulfide ligands have been considerably studied in the field of supramolecular chemistry over past years (Horikoshi & Mochida, 2006). Herein, we report the molecular structure of the title compound (I) - the newly synthesized disulfide ligand.

In (I) (Fig. 1), the C—S—S—C torsion angle of 96.12 (9)° is much larger than that in its analogue, namely 2,2'-dithiobis(4-pyridin-3-yl-pyrimidine) (Ji *et al.*, 2009).The S—S bond length of 2.0239 (8) Å in  (I) is within the normal range (Higashi *et al.*, 1978; Tabellion *et al.*, 2001). In the crystal, molecules are linked into chains through intermolecular aromatic π-π interactions (Table 1) .

### Experimental

A solution of SO_2_Cl_2_ (0.5 ml) in CH_2_Cl_2_ (20 ml) was added dropwise into the suspension containing 4-(pyridin-4-yl)pyrimidine-2-thiol (1.89 g) and 30 ml of CH_2_Cl_2_. Upon addition, the mixture was stirred at room temperature for 30 min. The solid was collected by filtration and dissolved into 30 ml of H_2_O. The solution PH was adjusted into the range of 8–9 to give white precipitates. Single crystals suitable for X-ray diffraction analysis were obtained by slow evaporation of the CH_2_Cl_2_ solution of the title compound.

### Refinement

All H atoms were positioned geometrically and allowed to ride on their parent atoms, with C—H = 0.93 Å and *U*iso(H) = 1.2*U*eq(C).

### Crystal data

**Table:**

C_18_H_12_N_6_S_2_	*Z* = 2
*M**_r_* = 376.48	*F*(000) = 388
Triclinic, *P*1	*D*_x_ = 1.452 Mg m^−^^3^
Hall symbol: -P 1	Mo *K*α radiation, λ = 0.71073 Å
*a* = 9.1060 (8) Å	Cell parameters from 3982 reflections
*b* = 9.3861 (9) Å	θ = 2.3–25.5°
*c* = 10.9176 (10) Å	µ = 0.32 mm^−^^1^
α = 84.228 (1)°	*T* = 298 K
β = 74.926 (1)°	Block, yellow
γ = 72.983 (1)°	0.14 × 0.12 × 0.10 mm
*V* = 861.27 (14) Å^3^	

### Data collection

**Table:**

Bruker APEXII CCD area-detector diffractometer	3982 independent reflections
Radiation source: fine-focus sealed tube	3283 reflections with *I* > 2σ(*I*)
graphite	*R*_int_ = 0.093
φ and ω scans	θ_max_ = 28.3°, θ_min_ = 1.9°
Absorption correction: multi-scan (*SADABS*; Bruker, 2001)	*h* = −11→12
*T*_min_ = 0.884, *T*_max_ = 0.920	*k* = −10→11
5665 measured reflections	*l* = −14→13

### Refinement

**Table:**

Refinement on *F*^2^	Primary atom site location: structure-invariant direct methods
Least-squares matrix: full	Secondary atom site location: difference Fourier map
*R*[*F*^2^ > 2σ(*F*^2^)] = 0.056	Hydrogen site location: inferred from neighbouring sites
*wR*(*F*^2^) = 0.158	H-atom parameters constrained
*S* = 1.12	*w* = 1/[σ^2^(*F*_o_^2^) + (0.092*P*)^2^] where *P* = (*F*_o_^2^ + 2*F*_c_^2^)/3
3982 reflections	(Δ/σ)_max_ = 0.026
235 parameters	Δρ_max_ = 0.63 e Å^−^^3^
0 restraints	Δρ_min_ = −0.50 e Å^−^^3^

### Special details

**Table:**

Geometry. All e.s.d.'s (except the e.s.d. in the dihedral angle between two l.s. planes) are estimated using the full covariance matrix. The cell e.s.d.'s are taken into account individually in the estimation of e.s.d.'s in distances, angles and torsion angles; correlations between e.s.d.'s in cell parameters are only used when they are defined by crystal symmetry. An approximate (isotropic) treatment of cell e.s.d.'s is used for estimating e.s.d.'s involving l.s. planes.
Refinement. Refinement of *F*^2^ against ALL reflections. The weighted *R*-factor *wR* and goodness of fit *S* are based on *F*^2^, conventional *R*-factors *R* are based on *F*, with *F* set to zero for negative *F*^2^. The threshold expression of *F*^2^ > σ(*F*^2^) is used only for calculating *R*-factors(gt) *etc*. and is not relevant to the choice of reflections for refinement. *R*-factors based on *F*^2^ are statistically about twice as large as those based on *F*, and *R*- factors based on ALL data will be even larger.

### Fractional atomic coordinates and isotropic or equivalent isotropic displacement parameters (Å^2^)

**Table:**

	*x*	*y*	*z*	*U*_iso_*/*U*_eq_	
S1	0.17317 (7)	0.15307 (5)	0.45673 (4)	0.04865 (18)	
S2	0.04746 (6)	0.10746 (6)	0.34511 (5)	0.05008 (19)	
N5	0.33888 (18)	−0.04716 (16)	0.21420 (13)	0.0350 (3)	
N3	0.23093 (18)	0.39811 (16)	0.49060 (14)	0.0390 (3)	
C13	0.4305 (2)	−0.12822 (18)	0.11230 (16)	0.0350 (4)	
C10	0.1865 (2)	0.00258 (19)	0.21737 (17)	0.0372 (4)	
C14	0.6028 (2)	−0.18393 (19)	0.10565 (16)	0.0359 (4)	
N4	0.1100 (2)	−0.01902 (19)	0.13474 (17)	0.0465 (4)	
N2	0.1638 (2)	0.39133 (19)	0.29373 (16)	0.0488 (4)	
C18	0.7094 (2)	−0.2658 (2)	0.00391 (19)	0.0450 (4)	
H18A	0.6733	−0.2889	−0.0618	0.054*	
C9	0.1901 (2)	0.3355 (2)	0.40474 (17)	0.0391 (4)	
C15	0.6663 (2)	−0.1528 (2)	0.19982 (18)	0.0445 (4)	
H15A	0.6009	−0.0976	0.2695	0.053*	
C6	0.2505 (2)	0.53454 (19)	0.45950 (17)	0.0382 (4)	
C5	0.2986 (2)	0.6052 (2)	0.55342 (17)	0.0398 (4)	
N6	0.9295 (2)	−0.2845 (2)	0.09198 (18)	0.0547 (5)	
C1	0.3575 (2)	0.7282 (2)	0.5210 (2)	0.0477 (5)	
H1A	0.3689	0.7692	0.4391	0.057*	
C17	0.8683 (3)	−0.3123 (3)	0.0009 (2)	0.0537 (5)	
H17A	0.9372	−0.3662	−0.0684	0.064*	
C11	0.2021 (2)	−0.0998 (2)	0.03537 (19)	0.0481 (5)	
H11A	0.1556	−0.1189	−0.0254	0.058*	
C7	0.2231 (3)	0.6050 (2)	0.3465 (2)	0.0510 (5)	
H7A	0.2337	0.7005	0.3253	0.061*	
C12	0.3641 (2)	−0.1563 (2)	0.01909 (19)	0.0445 (4)	
H12A	0.4265	−0.2112	−0.0517	0.053*	
C16	0.8274 (3)	−0.2050 (3)	0.1883 (2)	0.0525 (5)	
H16A	0.8673	−0.1831	0.2522	0.063*	
C4	0.2837 (3)	0.5510 (3)	0.6763 (2)	0.0586 (6)	
H4B	0.2450	0.4686	0.7018	0.070*	
N1	0.3860 (3)	0.7375 (2)	0.7322 (2)	0.0695 (6)	
C8	0.1796 (3)	0.5286 (2)	0.2664 (2)	0.0546 (5)	
H8A	0.1605	0.5747	0.1904	0.066*	
C2	0.3989 (3)	0.7884 (3)	0.6135 (2)	0.0584 (6)	
H2B	0.4387	0.8704	0.5905	0.070*	
C3	0.3270 (4)	0.6206 (3)	0.7623 (2)	0.0737 (8)	
H3B	0.3142	0.5837	0.8455	0.088*	

### Atomic displacement parameters (Å^2^)

**Table:**

	*U*^11^	*U*^22^	*U*^33^	*U*^12^	*U*^13^	*U*^23^
S1	0.0729 (4)	0.0372 (3)	0.0380 (3)	−0.0224 (2)	−0.0072 (2)	−0.0058 (2)
S2	0.0442 (3)	0.0486 (3)	0.0559 (3)	−0.0174 (2)	0.0021 (2)	−0.0188 (2)
N5	0.0447 (8)	0.0311 (7)	0.0309 (7)	−0.0148 (6)	−0.0068 (6)	−0.0009 (6)
N3	0.0498 (9)	0.0321 (7)	0.0342 (7)	−0.0121 (6)	−0.0061 (6)	−0.0053 (6)
C13	0.0472 (9)	0.0291 (8)	0.0291 (8)	−0.0153 (7)	−0.0047 (7)	−0.0005 (6)
C10	0.0462 (10)	0.0306 (8)	0.0372 (9)	−0.0179 (7)	−0.0061 (7)	−0.0008 (7)
C14	0.0467 (10)	0.0302 (8)	0.0311 (8)	−0.0141 (7)	−0.0065 (7)	0.0013 (6)
N4	0.0465 (9)	0.0481 (9)	0.0506 (9)	−0.0194 (7)	−0.0123 (7)	−0.0064 (7)
N2	0.0622 (11)	0.0463 (9)	0.0425 (9)	−0.0183 (8)	−0.0164 (8)	−0.0035 (7)
C18	0.0487 (11)	0.0450 (10)	0.0391 (9)	−0.0091 (8)	−0.0082 (8)	−0.0087 (8)
C9	0.0438 (9)	0.0359 (9)	0.0345 (8)	−0.0099 (7)	−0.0032 (7)	−0.0071 (7)
C15	0.0516 (11)	0.0480 (11)	0.0344 (9)	−0.0162 (8)	−0.0078 (8)	−0.0036 (8)
C6	0.0430 (9)	0.0324 (8)	0.0372 (9)	−0.0090 (7)	−0.0062 (7)	−0.0047 (7)
C5	0.0448 (10)	0.0307 (8)	0.0414 (9)	−0.0081 (7)	−0.0061 (8)	−0.0084 (7)
N6	0.0498 (10)	0.0580 (11)	0.0529 (10)	−0.0117 (8)	−0.0121 (8)	0.0037 (8)
C1	0.0515 (11)	0.0438 (10)	0.0470 (11)	−0.0161 (8)	−0.0045 (8)	−0.0085 (8)
C17	0.0528 (12)	0.0509 (12)	0.0489 (11)	−0.0052 (9)	−0.0055 (9)	−0.0087 (9)
C11	0.0554 (11)	0.0548 (12)	0.0429 (10)	−0.0236 (9)	−0.0148 (9)	−0.0085 (9)
C7	0.0694 (13)	0.0378 (10)	0.0502 (11)	−0.0189 (9)	−0.0198 (10)	0.0054 (8)
C12	0.0541 (11)	0.0462 (11)	0.0367 (9)	−0.0179 (8)	−0.0092 (8)	−0.0101 (8)
C16	0.0558 (12)	0.0634 (13)	0.0444 (11)	−0.0225 (10)	−0.0180 (9)	0.0042 (9)
C4	0.0924 (17)	0.0458 (12)	0.0475 (12)	−0.0306 (11)	−0.0207 (11)	−0.0032 (9)
N1	0.0925 (16)	0.0651 (13)	0.0639 (13)	−0.0318 (11)	−0.0235 (12)	−0.0193 (10)
C8	0.0737 (14)	0.0501 (12)	0.0465 (11)	−0.0202 (10)	−0.0255 (11)	0.0069 (9)
C2	0.0635 (14)	0.0516 (13)	0.0656 (15)	−0.0244 (10)	−0.0103 (11)	−0.0161 (11)
C3	0.120 (2)	0.0663 (16)	0.0496 (13)	−0.0387 (16)	−0.0302 (15)	−0.0036 (11)

### Geometric parameters (Å, °)

**Table:**

S1—C9	1.7867 (19)	C5—C4	1.374 (3)
S1—S2	2.0238 (7)	C5—C1	1.388 (3)
S2—C10	1.7734 (19)	N6—C17	1.339 (3)
N5—C10	1.321 (2)	N6—C16	1.331 (3)
N5—C13	1.352 (2)	C1—C2	1.385 (3)
N3—C9	1.335 (2)	C1—H1A	0.9300
N3—C6	1.342 (2)	C17—H17A	0.9300
C13—C12	1.392 (2)	C11—C12	1.384 (3)
C13—C14	1.486 (3)	C11—H11A	0.9300
C10—N4	1.336 (2)	C7—C8	1.382 (3)
C14—C18	1.393 (3)	C7—H7A	0.9300
C14—C15	1.395 (3)	C12—H12A	0.9300
N4—C11	1.332 (3)	C16—H16A	0.9300
N2—C8	1.333 (3)	C4—C3	1.389 (3)
N2—C9	1.323 (2)	C4—H4B	0.9300
C18—C17	1.377 (3)	N1—C2	1.323 (3)
C18—H18A	0.9300	N1—C3	1.335 (3)
C15—C16	1.380 (3)	C8—H8A	0.9300
C15—H15A	0.9300	C2—H2B	0.9300
C6—C7	1.386 (3)	C3—H3B	0.9300
C6—C5	1.491 (2)		
			
Cg1···Cg2^i^	3.888 (2)	Cg3···Cg4^ii^	3.572 (1)
			
C9—S1—S2	104.02 (6)	C2—C1—C5	118.5 (2)
C10—S2—S1	106.81 (6)	C2—C1—H1A	120.8
C10—N5—C13	115.68 (14)	C5—C1—H1A	120.8
C9—N3—C6	115.86 (16)	N6—C17—C18	123.76 (19)
N5—C13—C12	120.70 (17)	N6—C17—H17A	118.1
N5—C13—C14	116.75 (15)	C18—C17—H17A	118.1
C12—C13—C14	122.55 (16)	N4—C11—C12	122.50 (16)
N5—C10—N4	128.71 (17)	N4—C11—H11A	118.8
N5—C10—S2	122.14 (13)	C12—C11—H11A	118.7
N4—C10—S2	109.10 (13)	C8—C7—C6	117.84 (19)
C18—C14—C15	116.68 (17)	C8—C7—H7A	121.1
C18—C14—C13	121.97 (16)	C6—C7—H7A	121.1
C15—C14—C13	121.33 (16)	C11—C12—C13	117.66 (17)
C10—N4—C11	114.73 (16)	C11—C12—H12A	121.2
C8—N2—C9	114.58 (16)	C13—C12—H12A	121.2
C17—C18—C14	119.77 (18)	N6—C16—C15	124.43 (18)
C17—C18—H18A	120.1	N6—C16—H16A	117.8
C14—C18—H18A	120.1	C15—C16—H16A	117.8
N3—C9—N2	128.50 (18)	C5—C4—C3	119.3 (2)
N3—C9—S1	111.00 (14)	C5—C4—H4B	120.4
N2—C9—S1	120.50 (14)	C3—C4—H4B	120.4
C16—C15—C14	119.14 (18)	C2—N1—C3	116.0 (2)
C16—C15—H15A	120.4	N2—C8—C7	122.63 (19)
C14—C15—H15A	120.4	N2—C8—H8A	118.7
N3—C6—C7	120.54 (17)	C7—C8—H8A	118.7
N3—C6—C5	116.60 (16)	N1—C2—C1	124.7 (2)
C7—C6—C5	122.84 (17)	N1—C2—H2B	117.6
C4—C5—C1	117.73 (18)	C1—C2—H2B	117.6
C4—C5—C6	120.55 (18)	N1—C3—C4	123.8 (2)
C1—C5—C6	121.71 (18)	N1—C3—H3B	118.1
C17—N6—C16	116.21 (18)	C4—C3—H3B	118.1

Symmetry codes: (i) −*x*+1, −*y*+1, −*z*+1; (ii) −*x*+1, −*y*, −*z*.
